# Specific Colon Cancer Cell Cytotoxicity Induced by Bacteriophage *E* Gene Expression under Transcriptional Control of Carcinoembryonic Antigen Promoter

**DOI:** 10.3390/ijms160612601

**Published:** 2015-06-04

**Authors:** Ana R. Rama, Rosa Hernandez, Gloria Perazzoli, Miguel Burgos, Consolación Melguizo, Celia Vélez, Jose Prados

**Affiliations:** 1 Department of Health Science, University of Jaén, Jaén 23071, Spain; 2 Institute of Biopathology and Regenerative Medicine (IBIMER), Granada 18100, Spain; E-Mails: r_faraya@hotmail.com (R.H.); gperazzoli@ugr.es (G.P.); melguizo@ugr.es (C.M.); mariaceliavelez@ugr.es (C.V.); jcprados@ugr.es (J.P.); 3 Biosanitary Institute of Granada (ibs.GRANADA), SAS-University of Granada, Granada 18071, Spain; 4 Department of Human Anatomy and Embryology, School of Medicine, University of Granada, Granada 18012, Spain; 5 Institute of Biotechnology and Department of Genetics, University of Granada, Granada 18071, Spain; E-Mail: mburgos@ugr.es

**Keywords:** carcinoma embryonic antigen, colorectal cancer, *E* gene, suicide gene therapy, promoter tissue specific

## Abstract

Colorectal cancer is one of the most prevalent cancers in the world. Patients in advanced stages often develop metastases that require chemotherapy and usually show a poor response, have a low survival rate and develop considerable toxicity with adverse symptoms. Gene therapy may act as an adjuvant therapy in attempts to destroy the tumor without affecting normal host tissue. The bacteriophage *E* gene has demonstrated significant antitumor activity in several cancers, but without any tumor-specific activity. The use of tumor-specific promoters may help to direct the expression of therapeutic genes so they act against specific cancer cells. We used the carcinoembryonic antigen promoter (*CEA*) to direct *E* gene expression (*pCEA*-*E*) towards colon cancer cells. *pCEA*-*E* induced a high cell growth inhibition of human HTC-116 colon adenocarcinoma and mouse MC-38 colon cancer cells in comparison to normal human CCD18co colon cells, which have practically undetectable levels of CEA. In addition, *in vivo* analyses of mice bearing tumors induced using MC-38 cells showed a significant decrease in tumor volume after *pCEA*-*E* treatment and a low level of Ki-67 in relation to untreated tumors. These results suggest that the *CEA* promoter is an excellent candidate for directing E gene expression specifically toward colon cancer cells.

## 1. Introduction

Colon cancer, along with breast and lung cancer, is one of the most prevalent cancers in the world [1]. While in early stages, colon cancer is characterized by a good prognosis, in more advanced, metastatic stages, the five-year survival rate is only 10%. Approximately 25% of all colon cancer patients reach this stage and are principally treated with 5-fluorouracil (5-FU) alone or a combination of oxaliplatin (FOLFOX, a combo of oxaliplatin, 5-FU and leucovorin), irinotecan (FOLFIRI, a combo of irinotecan, 5-FU and leucovorin), angiogenesis inhibitors and/or epidermal growth factor receptor inhibitors [2]. However, the results from current treatments are poor and may be accompanied by tissue damage. In this context, gene therapy tries to modify or destroy the tumor cell uniquely from within, without causing damage to any other tissues. Recent studies have investigated several aspects of gene therapy related to cancer treatment; one of these approaches is suicide gene therapy [3], which may enhance the potential of the drugs typically used to treat cancer [4], including colon cancer [5,6].

Classic systems of suicide gene therapy rely on the administration of a prodrug. The prodrug is catalyzed by suicide enzymes to produce a toxic substance capable of inducing cancer cell death. The most representative enzyme of this therapeutic strategy, thymidine kinase (TK), has been assayed in clinical trials against gliomas [7], prostate cancer [8] and hepatocellular carcinoma [9], among others. However, the conversion of a non-toxic prodrug into toxic metabolites and the bioavailability of the activated drug severely limit the system’s efficacy. These causes of treatment failure are currently overcome by using genes that encode for cytotoxic proteins, which have a direct antitumor action. Some of these genes are taken from non-eukaryotic organisms, such as viruses, bacteria and plants [4,10,11,12]. We have recently shown how the toxic *E* gene from the bacteriophage ϕX174, which codes for a 91-amino acid membrane protein with lytic function [6,13,14], significantly decreased colon cancer cell proliferation, inducing mitochondrial apoptosis. Analysis of the mechanism suggests the formation of a “transmembrane pore” through which the cell loses cytoplasmic content. Interestingly, this gene did not need a prodrug to induce cell death [15]. The use of tumor-specific promoters that are overexpressed in cancer could drive transcription of these proteins known to be selectively active in tumor cells, thus obtaining a therapeutic system with a more specifically localized activity. Recently, survivin promoter [16], human telomerase reverse transcriptase promoter [17] and epithelial cell adhesion molecule (EpCAM) promoter [18] have been assayed to delivery *TK* or *CD* (*cytosine deaminase*) genes in cancer cells.

Carcinoma embryonic antigen (CEA) is an oncofetal tumor marker, which is overexpressed in over 90% of colorectal cancer cells, but not in normal colon cells [19,20,21]. In addition, CEA can be detected in 70% of colorectal cancer diagnoses [22,23]. Michl *et al.* [24] discovered significantly elevated CEA serum concentrations in patients in the final stages of the pathology; hence, they used CEA as a prognosis marker. Shibutani *et al.* [22] corroborated the utility of CEA levels for predicting the prognosis and also for monitoring recurrence and metastasis after potentially curative surgery in patients with stage II colorectal cancer. Wang *et al.* [25] concluded that high levels of tissue mRNA expression and CEA serum are associated with the incidence and progression of colorectal cancer, while Patel *et al.* [26] used CEA as a clinical and pathologic prognostic marker of local recurrence and overall survival after resection. Thus, the *CEA* promoter has been used in gene therapy to direct the expression of therapeutic genes toward CEA-positive cancer cells [16]. In fact, Zhang *et al.* [27] demonstrated the selective expression, under the transcriptional control of the *CEA* promoter, of the cytosine deaminase (CD) enzyme in colon cancer cells.

The aim of this study was to investigate the activity of the *E* gene, a toxic gene for colon cancer cells, under *CEA* promoter transcriptional control, which is frequently overexpressed in this type of tumor cell. We analyzed different colon cancer cell lines in order to select those with differential CEA expression. Colon cancer cells were then transfected *in vitro* to assess the anti-proliferative effect of the *E* gene under the influence of the *CEA* promoter. We also analyzed the *in vivo* cytotoxicity of the *pCEA*-*E* gene to demonstrate its activity with respect to tumor growth and mouse survival rates. Furthermore, we analyzed the toxicity of this new gene therapy and its effect over proliferation rates.

## 2. Results

### 2.1. Transcriptional Activity of CEA Promoter

**Figure 1:**
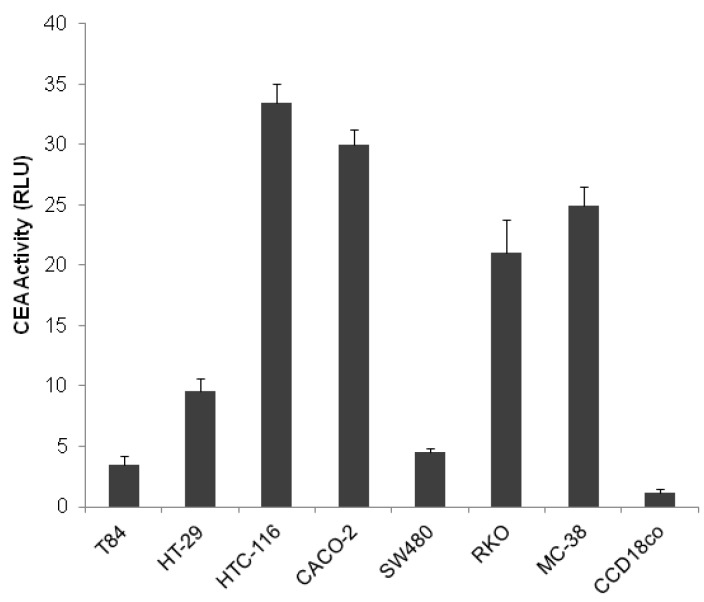
Transcriptional activity of CEA. Human CACO-2, HT29, HCT-116, SW480, RKO and T-84 colon adenocarcinoma cell lines, mice MC-38 colon cancer cell line, and the normal human colon CCD18co cell line Cells were co-transfected with luciferase expression vectors pPGL2/CEA or pGL2 and the Renilla expression vector CMV/Renilla. Luciferase activity of each transfection was normalized by the Renilla reading. Luciferase activity is represented by the ratio of the specific promoter over the activity of pGL2. Data represent the mean value of three replicates ± the standard error of the mean (SEM).

*CEA* activity was detected in all seven colon cancer cell lines by luciferase assay. Human HTC-116, CACO-2, RKO colon adenocarcinoma cell lines and mouse MC-38 colon cancer cells showed high levels of luciferase expression, whereas human HT-29, T-84 and SW480 adenocarcinoma cell lines showed a low degree of luciferase expression. Specifically, HTC-116 showed the highest level of luciferase among all of the colorectal cancer cells, while T-84 cells presented the least expression of cancer cells. In contrast, normal intestinal epithelial CCD18co cells demonstrated the lowest levels of *CEA* activity (Figure 1).

### 2.2. In Vitro Inhibition of Cell Growth by pCEA-E

**Figure 2:**
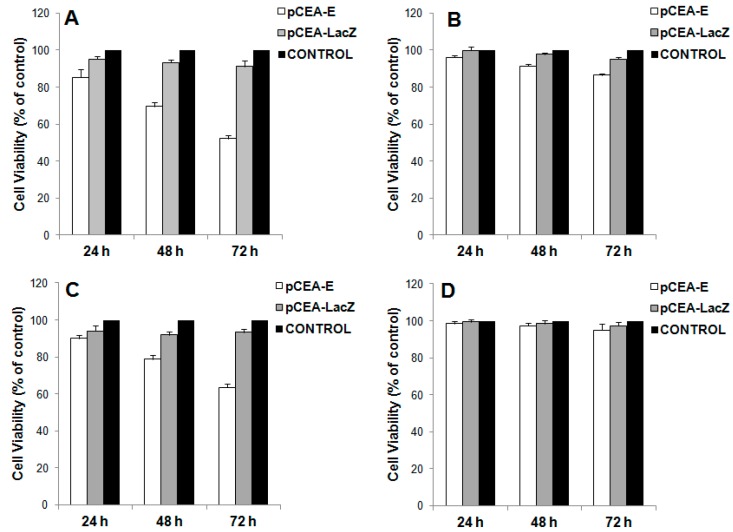
Effect of *pCEA*-*E* on cell proliferation. Cells from cell lines HTC-116 (**A**), T-84 (**B**), MC-38 (**C**) and CCD18co (**D**) were transfected with *pCEA*-*E* to determine proliferation rate modulation after 24, 48 and 72 h. Data represent the mean value of three replicates ± the standard error of the mean (SEM).

To analyze the *E* gene antiproliferative effect under *CEA* promoter transcriptional control, we selected the colon cancer cell lines with the highest (HTC-116) and lowest (T-84) *CEA* promoter activity, as well as normal CCD18co cells, which presented practically null *CEA* promoter activity. Furthermore, we analyzed MC-38 cells in order to carry out *in vivo* experiments. As shown in Figure 2A, HTC-116 cells transfected with the *E* gene showed a significant and time-dependent decrease in growth. Cell growth inhibition was 15%, 31% and 48% at 24, 48 and 72 h, respectively, after transfection and in relation to control cells. Similar results were observed for the MC-38 colon cancer cells, which were also characterized by strong *CEA* promoter activity (Figure 2C). By contrast, T-84 cells with weak *CEA* promoter activity showed proliferation inhibition levels of only 4%, 8.5% and 13.5% at 24, 48 and 72 h after transfection, respectively. CCD118co colon cells with no *CEA* promoter activity showed an insignificant change in the % of proliferation (see “control”). 

**Figure 3:**
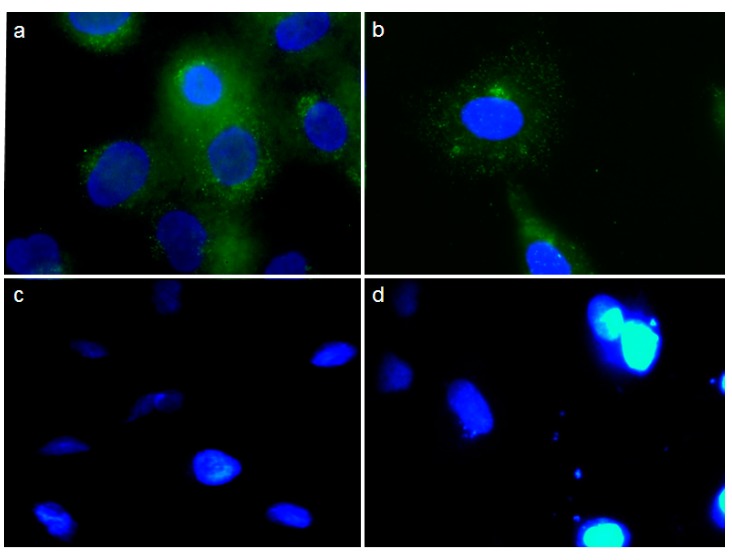
Subcellular localization of E/V5 fusion protein. Recombinant E protein (E-V5) was detected using anti-V5-FITC antibody (green) in HTC-116 (**a**) and MC-38 (**b**). No stain was observed in T-84 (**c**) and CCD18co (**d**) cells following *pCEA*-*E* treatment. Twenty-four hours after transfection, the fluorescence pattern was dotted and located in the cell cytoplasm. Cell nuclei were stained by DAPI (blue). Magnification: 40×.

Expression of E protein in cells transfected with *pCEA*-*E* was confirmed using the anti-V5-FITC antibody. Positive staining was detected in HTC-116 and MC-38 cells, but not in T-84 and CCD118co cells (Figure 3).

### 2.3. In Vivo Tumor Growth Inhibition and Survival Analysis

**Figure 4:**
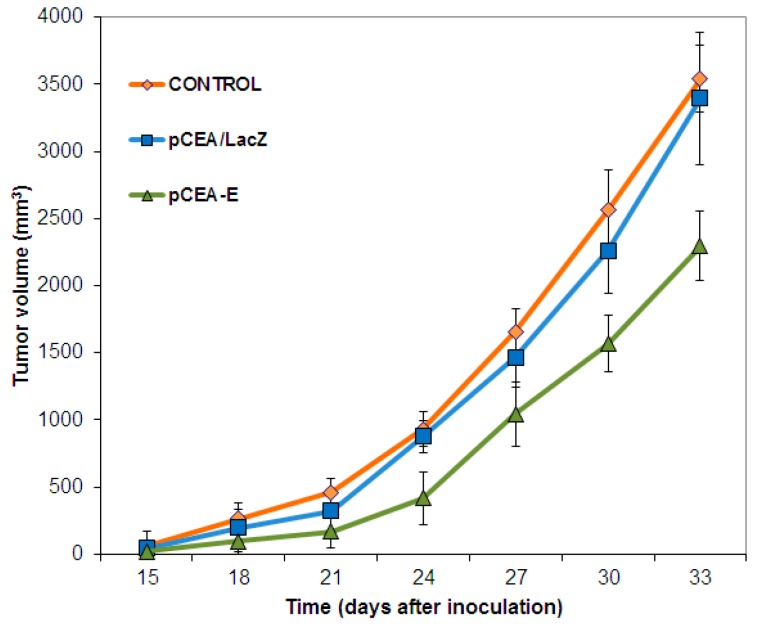
Tumor growth inhibition induced by *pCEA*-*E*. Treatment with *pCEA*-*E* induced a significant reduction in tumor volume at the end of the study period (33 days) in comparison with the growth of *pCEA*-*LacZ*-treated or untreated tumors (control) (*p* < 0.05). Data represent the mean value ± SEM.

As Figure 4 shows, intratumoral treatment with *pCEA-E* produced significant reductions in tumor volumes after 33 days. Specifically, *pCEA-E* was able to induce a 36% tumor volume reduction in comparison to the control (*p <* 0.05). *pCEA-LacZ* treatment, on the other hand, did not bring about any modifications in tumor growth rates, yielding similar results to those observed in the untreated mouse group. Nevertheless, although *pCEA-E* treatment produced reductions in tumor volumes, it failed to increase mouse survival rates compared to the untreated control group (data not shown).

### 2.4. In Vivo Toxicity

**Figure 5:**
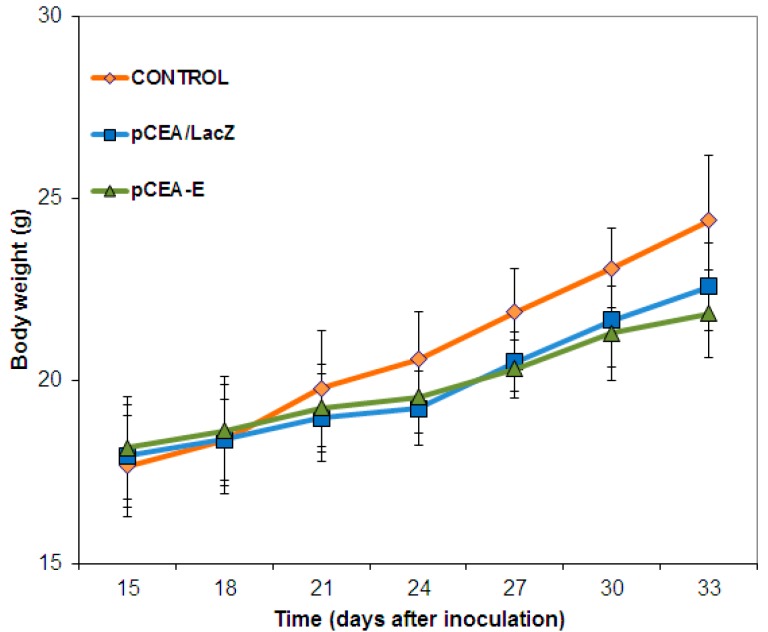
Weight evolution after *pCEA*-*E* treatment. The weights of mice bearing subcutaneous MC-38 tumors and treated with *pCEA*-*E* were measured. *pCEA*-*E*-treated mice experienced a similar weight evolution to that of *pCEA*-*LacZ*-treated and untreated mice (control). Data represent the mean value ± SEM.

Mouse body mass was monitored throughout the treatment in order to determine the *in vivo* toxicity of the *pCEA-E* transfected gene. Both mice treated with *pCEA-E* or *pCEA*-*LacZ* revealed no significant weight loss in comparison to the control group, suggesting that neither of the plasmid administrations induced any toxicity (Figure 5).

### 2.5. Effect of pCEA-E on Cell Proliferation and Apoptosis

**Figure 6:**
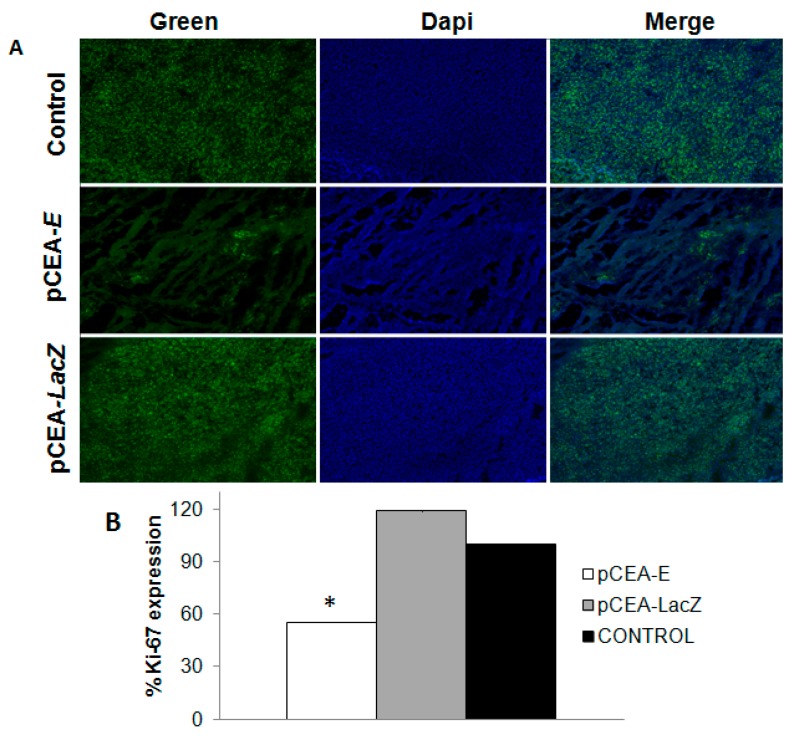
Immunohistochemical staining of Ki-67 expression in mice bearing subcutaneous MC-38 tumors treated with *pCEA*-*E*. (**A**) Images of Ki-67 expression (green) and cell nuclei stained with DAPI (blue). Original magnification: 10×; and (**B**) Ki-67 histogram protein expression profiles. Ki-67 expression was lower in the *pCEA*-*E*-treated group than in the *pCEA*-*LacZ* and control groups (* *p* < 0.05).

To determine the effect of *pCEA-E* on cell proliferation and apoptosis, we examined Ki-67 expression using immunohistochemical staining and detected apoptotic cells using the TUNEL assay. Our results showed that Ki-67 expression significantly decreased (55.4% of expression relative to the control tissue) in tumors that received *pCEA-E* treatment, suggesting significant inhibition of cell proliferation (*p <* 0.05) (Figure 6).

**Figure 7:**
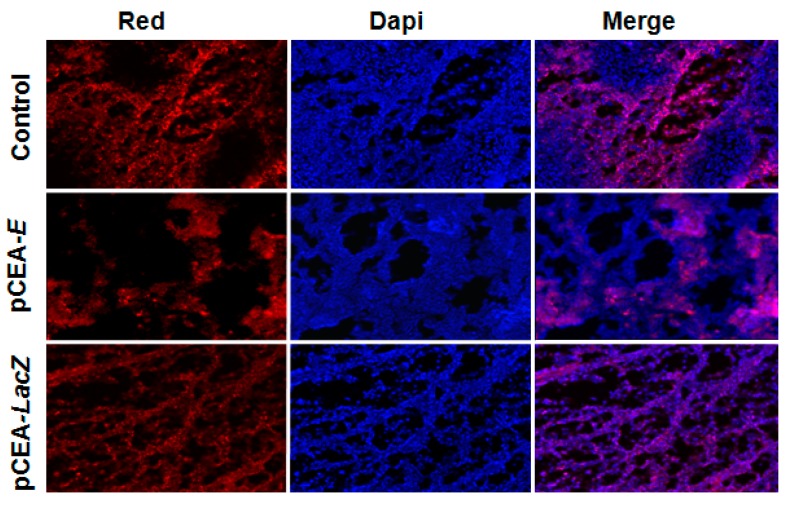
TUNEL assay in mice bearing subcutaneous MC-38 tumors. Images presenting apoptotic cells (red) and nuclei stained by DAPI (blue). *pCEA*-*E* treatment did not lead to an increased number of apoptotic cells in comparison with *pCEA*-*LacZ* or untreated tumors (control). Original magnification: 20×.

By contrast, the *pCEA*-*LacZ* treatment did not modulate Ki-67 expression, which was similar to the control group. On the other hand, analysis of the apoptosis-linked DNA fragmentation using a TUNEL assay showed similar staining between tumors treated with *pCEA-E* and the control tissue (Figure 7).

## 3. Discussion

Despite significant advances in the development of new therapies and improvements in traditional treatments for patients with advanced colon cancer, the prognosis and survival rate for these patients remains poor. New gene therapy strategies are being developed to treat cancers, but to date have only demonstrated low levels of *in vivo* efficacy in terms of gene delivery and tumor specificity [17,28]. The utility of tumor-specific promoters for gene targeted expression has been described in various cancers, such as melanoma, brain, lung, pancreas and colon [16,21,27,29,30]. Our study described the use of the *CEA* promoter to increase the colon cancer cell specificity of *E* gene expression and the subsequent tumor growth inhibition induced by expressing this cytotoxic gene.

We have previously shown that *E* gene expression can induce apoptosis, dilated mitochondria with disrupted cristae, caspase-3 activation and the release of cytochrome C into the cytoplasm of some cancer cells [5,6,13]. In this study, we analyzed CEA activity through luciferase expression in several cell lines. We found the highest expression among all colon cancer cells in HTC-116 cell lines (33.4%) and the lowest in T-84 cells (3.49%); whereas normal CCD18co cells revealed practically undetectable levels of CEA. These results for *CEA* promoter activity are similar to those described by Gou *et al.* [31] and Dąbrowska *et al.* [32], who also observed a greater *CEA* promoter activity in colon cancer cell lines than in non-tumor cancer cell lines using a luciferase assay.

Analysis of luciferase expression was correlated against the *E* gene’s inhibitor effect and revealed a decrease in the proliferation according to HTC-116 > MC-38 > T-84 > CCD18co. Furthermore, *E* gene transfection induced a significant and time-dependent decrease in HTC-116 and MC-38 cell growth, as has occurred in other cancer cells lines [6,13,14]. In fact, the greatest level of growth inhibition was found in both HTC-116 cells and MC-38 cells 72 h after transfection (48% and 36.6%, respectively). By contrast, low levels of growth inhibition were detected in T-84 and CCD18co cells (13.6% and 5.1% at 72 h after transfection, respectively). Other experiments using suicide genes under *CEA* transcriptional expression control corroborated a specific growth inhibition in cells with high levels of CEA expression. In fact, Zhang *et al.* [27] studied the effect of the *CD* suicide gene under *CEA* promoter control in the LoVo human colon cancer cell line (CEA-positive) in comparison to HeLa cells (a CEA-negative human adenocarcinoma cell line). Both cell lines were transfected with CEA-*CD* and treated with 5-FC for five days. HeLa cells transfected with CEA-*CD* did not sensitize the cytotoxicity caused by 5-FC, whereas CEA-*CD* LoVo cells showed a 72.7% growth inhibition. A similar study by Liu *et al.* [33] revealed a greater inhibition of growth (89.8%) in a CEA-positive human gastric cancer cell line (SGC7901 cells) than in a HeLa cell line (2%); in this case, both lines were treated with a double suicide gene system (*TK* and *CD* genes*)* under CEA control.

To demonstrate the *in vivo* efficiency of our therapeutic system, we investigated the activity of the *CEA* promoter-*E* gene in mice bearing colon cancer tumors from MC-38 CEA-positive cells. Statistical evaluation of tumor growth rates obtained from mice treated with *pCEA-E* revealed a significant decrease (36%) of tumor volume in comparison to the control group. These results support the hypothesis that the *E* gene under *CEA* post-transcriptional control was able to reduce the proliferation rate of a tumor generated by CEA-positive cells. Studies by Zhang *et al.* [27] demonstrated a similar effect in LoVo mice xenografts treated with the *CD* gene under the *CEA* promoter. Liu *et al.* [33] studied a human xenograft gastric carcinoma during treatment with a double gene therapy system (*CD* and *TK* gene) for 36 days. A tumor growth inhibition of 54% was reported in treated tumors compared to control tumors. This greater decrease in tumor volume compared to our findings may be explained by the use of calcium phosphate nanoparticles (CPNP) that increase transfection efficiency and the use of a stable cell line to express therapeutic genes. Interestingly, analysis of Ki-67 showed a strong degree of expression in the untreated and *pCEA*-*LacZ* groups (controls) in relation to tumors intratumorally treated with the *pCEA-E* vector. The decreased level of Ki-67 expression in *pCEA*-*E* genes is representative of the colon cancer cells’ loss of proliferative capacity. In fact, Okabe *et al.* [34] have demonstrated that the *CEA* promoter in an adenovirus expressing the *TK* gene was able to improve its antitumor activity in mice bearing colorectal tumors from RCM-1 CEA-positive cells and to reduce the number of liver metastases (after 42 days of treatment) in relation to an untreated group of mice.

We previously reported the successful use of the *E* gene in cancer therapy. However, a lack of selective antitumor activity is its main limitation. The use of tumor-specific promoters of cytotoxic genes (but which have a low activity in normal cells) may direct therapeutic gene expression toward cancer cells. In this study, we demonstrated that the *CEA* promoter can induce *E* gene expression, which is selective of colon cancer cells, while the gene is practically unexpressed in normal cell lines. *E* gene expression under *CEA* control rapidly and efficiently inhibited cell growth in CEA-positive colon cancer cells, which, in turn, induced a significant decrease in the tumor volume of mice bearing such cancer cells. In addition, a decrease in Ki-67 tumor expression indicated a significant decrease in post-treatment cell proliferation. In conclusion, we propose that the *CEA* promoter provides a means of specifically directing *E* gene expression toward colon cancer cells in order to mediate suicide gene therapy.

## 4. Experimental Section

### 4.1. Cell Culture

The human colon adenocarcinoma CACO-2, HT29, HCT-116, SW480, RKO and T-84 cell lines, the mice colon cancer MC-38 cell line (kindly provided by Jeffrey Schlom from Public Health Service, National Institutes of Health, Bethesda, MD, USA) and the normal human colon CCD18co cell line (Instrumentation Service Center, University of Granada, Granada, Spain) were used in this study. All cell types were grown in Dulbecco’s Modified Eagle’s Medium (DMEM) (Sigma, St. Louis, MO, USA), supplemented with 10% fetal bovine serum (FBS) and 1% streptomycin-penicillin (Sigma), under air containing 5% CO_2_ and in an incubator at 37 °C.

### 4.2. Construction of Luciferase and E Expression Vectors

*CEA* promoter from human genomic DNA was amplified by PCR using the primers: *CTCGAG*CCATCCACCTTGCCGAAA and *AAGCTT*GCTGTCTGCTCTGTCCTC. *XhoI* and *HindIII* restriction sites were introduced into the forward and reverse primers, respectively. The *CEA* promoter fragment and pGL2 vector (Promega Biotech Ibérica, Madrid, Spain) were digested with *XhoI* and *HindIII* restriction enzymes, and the *CEA* promoter fragment was finally cloned in the pGL2 vector (pGL2/CEA). We used a previously developed vector, pcDNA3.1/E (Rama *et al.*, 2010), to construct the E expression vector under the control of the *CEA* promoter. Firstly, the *CEA* promoter was amplified by PCR from pGL2/CEA using the primers *CAATTG*CCATCCACCTTGCCGAAA and *GAGCTC*GCTGTCTGCTCTGTCCTC with *MfeI* and *SacI* restriction sites. Secondly, the *pcDNA3.1/E* *CMV* promoter was removed by digestion with *MfeI* and *SacI*. Finally, CEA was digested with the same restriction enzymes and ligated in place of the *CMV* promoter, thus generating pcDNA3.1/CEA/E (*pCEA-E*). pcDNA3.1/CEA/*LacZ* (*pCEA-LacZ*) was used as a control and generated from pcDNA3.1/*LacZ* using a method similar to the one for *pCEA-E* [13]. Subcloning Efficiency™ DH5α™-competent *E. coli* cells (Qiagen, Barcelona, Spain) were transformed with the generated plasmids and their correct sequences confirmed by DNA sequencing.

### 4.3. Luciferase Assay

All transfections were performed using FUGENE6 reagent (Roche Diagnostic, Barcelona, Spain) according to the manufacturer’s instructions. Cells (7 × 10^3^) were seeded into 96-well culture plates. After 24 h, the cells were co-transfected with 0.2 μg/well of luciferase reporter vectors (pGL2/CEA or pGL2) and 0.05 μg/well of CMV/Renilla vector as internal controls for normalization of the transcriptional activity of the reporter vectors (provided by Miguel Burgos of the Biotechnology Institute, Granada, Spain). Experiments were performed in three groups: cells transfected with pGL2/CEA and CMV/Renilla; cells transfected with pGL2 and CMV/Renilla; and untransfected cells (control). Forty-eight hours after transfection, luciferase activity was determined using the Dual-Glo Luciferase Assay System (Promega) according to the manufacturer’s instructions. The luminescence was measured in a luminometer (96 GloMax^®^ microplate). The luciferase activity in each well was normalized to CMV/Renilla using the formula *Ln* = *L*/*R* (*Ln*: Normalized luciferase activity; L: Luciferase activity reading; R: Renilla activity reading). The *Ln* was further standardized according to the transcriptional activity of pGL2 using the formula of *RLU* = *Ln*/pGL3-basic (RLU: Relative luciferase unit). All transfections were performed in triplicate.

### 4.4. In Vitro Proliferation Assay

To determine the rate of proliferation, transfection was performed using FUGENE6 reagent (Roche Diagnostic) as described previously. Cells were seeded in 24-well plates (10^4^ cells per well) to analyze proliferation. After 24 h, the cells were transfected with 1 μg/well of the respective vector (*pCEA-E* or *pCEA*-*LacZ*). Untransfected cells were used as a control. After 24, 48 and 72 h, MTT (3-(4,5-dimethylthiazol-2-yl)-2,5-diphenyltetrazolium bromide) solution (5 mg/mL) was added to each well (20 μL) and incubated for 4 h at 37 °C. Two hundred microliters of dimethylsulfoxide (DMSO) were then added to each well after the medium had been removed. Optical density was determined using a Titertek multiscan colorimeter (Flow Laboratories, Irvine, CA, USA) at 570 and 690 nm. Cells transfected with empty vectors were used as controls.

### 4.5. Microscopic Analysis

pcDNA3.1 provides a V5 epitope tag for efficient detection of recombinant proteins. Therefore, E protein expression was confirmed using the anti-V5-FITC antibody (Invitrogen, Madrid, Spain). The cells were grown on coverslips and transfected with *pCEA-E* and *pCEA-LacZ*. As above, untransfected cells were used as a control. After 24 h of transfection, cells were washed with PBS, fixed in 100% methanol at room temperature for 3 min, blocked with 10% bovine serum albumin/PBS for 20 min and then incubated with anti-V5-FIFC antibody diluted (1:500) in 1% bovine serum albumin/PBS. DAPI solution (100 nM) (Invitrogen, Madrid, Spain) was used for nuclear staining. The cells were then rinsed with PBS, mounted and visualized using fluorescent microscopy analysis (Nikon Eclipse Ti, Nikon Instruments Inc., Melville, NY, USA). V5 was excited at 488 nm and DAPI nuclear stain at 364 nm.

### 4.6. In Vivo Study

Female C57BL/6 mice (Scientific Instrumentation Centre, University of Granada, Granada, Spain) were used in the *in vivo* studies. All mice (body weight: 25–30 g) were kept in a laminar airflow cabinet located in an ambient-controlled room (37.0 ± 0.5 °C and a relative humidity of 40%–70%) and subjected to a 12-h day/night cycle under specific pathogen-free conditions. All studies were approved by the Ethics Committee of the Medical School at the University of Granada and complied with international standards (European Communities Council Directive 86/609). Tumors were induced by subcutaneous injection of 7 × 10^5^ MC-38 cells in 200 μL of PBS into the left flanks of mice. Tumors were allowed to grow to 75 mm^3^ (a minimal size for ideal intratumoral injection) before starting intratumoral treatment. Mice were randomly assigned to the following groups: treated with *pCEA-E*, treated with *pCEA-LacZ* and untreated (control). *In vivo* JetPEI (Polyplus-transfection Inc., New York, NY, USA) was used as a transfection enhancer reagent according to the manufacturer’s instructions. PEI/DNA complexes with a ratio of 1:6 were formed for 15 μg DNA/50 μL 10% glucose plus 1.8 μL *in vivo*-JetPEI/50 μL 10% glucose. Each mouse received intratumoral injections every three days, up to a maximum total of six administrations. Mice weights and deaths were recorded throughout this period. Tumor volumes (*V*, mm^3^) were estimated by using a digital caliper to measure the largest diameter “*a*” and the second largest diameter “*b*” perpendicular to “*a*”, then calculating the volume from, *V* = *ab*^2^ × π/6.

### 4.7. Histological Studies

Tumors were cryopreserved at −80 °C then cut into 15 μm-thick cryostat sections, collected on gelatin-coated slides and stored at −20 °C until used. The presence of apoptotic cells within the tumor sections was evaluated after 20 min of fixation with 4% paraformaldehyde at room temperature using the TUNEL technique with the In Situ Cell Death Detection Kit TMR red (Roche, Mannheim, Germany) according to the manufacturer’s recommendations. Cell nuclei were counterstained with DAPI and fluorescence images captured using a Nikon eclipse 50i microscope. Sections were probed with Ki-67(M-19) antibody (Santa Cruz Biotechnology Inc., Heidelberg, Germany) in order to evaluate proliferation. Tissue sections were fixed for 20 min with 4% paraformaldehyde at room temperature. The sections were then washed with PBS and blocked for 1 h with donkey serum before being incubated for 1 h at room temperature with the primary antibody (1:100). The sections were then washed with PBS and incubated at room temperature with an Alexa 488 anti-donkey secondary antibody (1:500) (Invitrogen, Madrid, Spain) for 1 h. Cell nuclei were again counterstained with DAPI and fluorescence images captured with a Nikon eclipse 50i microscope. Representatively, stained areas on all slides were digitally imaged, and TUNEL and Ki-67 protein expressions were quantified using ImageJ software plugins.

### 4.8. Statistical Analysis

All of the results were represented as the mean ± standard deviation (SEM). Statistical analysis was performed using the Student’s *t*-test (SPSS version 15, SPSS, Chicago, IL, USA). The probability of mice survival was determined by the Kaplan–Meier method, and the log-rank test was used to compare the fraction of surviving mice between groups (α = 0.05). Data with *p <* 0.05 and *p <* 0.001 were considered as significant and very significant, respectively.

## 5. Conclusions

New gene therapy strategies are being developed to treat cancers. The use of tumor-specific promoters are being developed may help to direct the expression of therapeutic genes so they act against specific cancer cells. We have proved that carcinoma embryonic antigen is an excellent tumor-specific promoter to direct *E* gene expression towards colon cancer cells but not to normal colon cells, inducing cell growth inhibition, Ki-67 expression reduced and decrease of tumor volume. We propose the system of the *E* gene under *CEA* post-transcriptional as a novel gene therapy strategy for the treatment of colorectal cancer.
